# Relationship Between Pain Intensity and Health-Related Quality of Life in Patients With Chronic Neck Pain: A Cross-Sectional Study

**DOI:** 10.7759/cureus.112960

**Published:** 2026-07-19

**Authors:** Kazi Mohd. Sayeeduzzaman, Md. Ahsan Ullah, AKM Salek, Kishoara Sultana

**Affiliations:** 1 Physical Medicine and Rehabilitation, Khwaja Yunus Ali Medical College and Hospital, Sirajgonj, BGD; 2 Physical Medicine and Rehabilitation, Bangabandhu Sheikh Mujib Medical University, Dhaka, BGD; 3 Dermatology and Venereology, Lion A. Badal Eye and General Hospital, Dhaka, BGD

**Keywords:** chronic neck pain, health-related quality of life, pain intensity, sf-36, visual analog scale

## Abstract

Background

Chronic neck pain (CNP) is a major contributor to disability worldwide and may also occur as part of widespread pain syndromes such as fibromyalgia. However, evidence regarding its impact on health-related quality of life (HRQoL) remains limited in low- and middle-income countries such as Bangladesh. This study aimed to assess the relationship between pain intensity and HRQoL among patients with CNP.

Materials and methods

A hospital-based cross-sectional study was conducted at Bangabandhu Sheikh Mujib Medical University in Dhaka from April 2021 to January 2022. A total of 96 adult patients (aged 20-60 years) with clinically diagnosed CNP (pain duration ≥3 months) were recruited using consecutive sampling. Pain intensity was measured using the visual analog scale (VAS). HRQoL was assessed using the Bangla version of the SF-36 questionnaire, which covers eight domains, as well as physical and mental health summary scores (PHS and MHS). Data were collected through face-to-face interviews and analyzed using SPSS Statistics version 25 (IBM Corp. Released 2017. IBM SPSS Statistics for Windows, Version 25.0. Armonk, NY: IBM Corp.). Pearson correlation coefficients were computed to evaluate associations between pain intensity and HRQoL domains.

Results

The mean age of the participants was 38.37 ± 10.28 years, and 71 (74.0%) were male in this hospital-based sample. The most impaired SF-36 domain was role limitation due to physical health (mean: 36.19 ± 22.02). Pain intensity showed a strong negative correlation with physical functioning (r = −0.713, p < 0.001) and moderate negative correlations with PHS (r = −0.420, p < 0.001), MHS (r = −0.527, p < 0.001), and total SF-36 score (r = −0.550, p < 0.001). No significant correlations were observed with social functioning (r = −0.131, p = 0.205) or mental health (r = −0.165, p = 0.109).

Conclusions

Higher pain intensity is significantly associated with poorer HRQoL, particularly affecting role limitation due to physical health and other physical and emotionally mediated domains in patients with CNP. Comprehensive rehabilitation strategies targeting pain reduction and functional restoration are urgently needed in Bangladesh.

## Introduction

Chronic neck pain (CNP) is recognized as a common and disabling musculoskeletal condition worldwide. The Global Burden of Disease (GBD) Study 2021 reported that neck pain affected nearly 203 million individuals globally in 2020, with an age-standardized prevalence of 2,450 per 100,000 population and an age-standardized years lived with disability (YLD) rate of 244 per 100,000 [1]. Trend data further indicate a marked rise in the global burden of neck pain, with prevalence increasing by 98.21% and YLDs by 78.17% between 1990 and 2019 [2]. The GBD 2021 estimates also suggest that the number of people affected by neck pain may increase to approximately 269 million by 2050, representing a 32.5% rise from 2020, largely due to population growth and aging [1]. As neck pain commonly affects working-age adults, it contributes substantially to absenteeism, reduced productivity, and increased healthcare costs. In the United States, neck pain combined with low back pain accounted for the highest healthcare expenditure in 2016, with spending estimated at $134.5 billion [3]. These findings highlight the growing clinical and public health importance of neck pain and support the need for detailed investigation into its determinants and consequences.

The etiology of CNP is multifactorial, encompassing mechanical, degenerative, and psychosocial factors [4]. Persistent pain may contribute to functional limitations and long-term disability [5].

CNP can adversely affect HRQoL across physical, psychological, and social domains [6]. Patients with nonspecific CNP have been reported to score below general population reference values across the 36-Item Short-Form Health Survey (SF-36) domains [7]. Pain duration and pain localization may also influence disability and health-related quality of life (HRQoL) [8]. Similarly, reduced quality of life has been reported among patients with rheumatoid arthritis who experience neck pain [9].

Pain intensity is a critical determinant of disability and HRQoL in patients with CNP [10]. Higher pain severity is consistently associated with worse functional outcomes and a diminished quality of life [8,10]. Studies utilizing the VAS to quantify pain intensity have demonstrated significant correlations with HRQoL measures [8,10]. For example, a cross-sectional study found that neck pain intensity, measured using the VAS, was negatively correlated with various quality-of-life domains in individuals with chronic nonspecific neck pain [10]. Another study assessing the effects of pain characteristics found a clear relationship between pain intensity at rest, during activity, and at sleep and various quality-of-life measures [8].

However, the strength of the association between pain intensity and HRQoL can vary. Some studies report moderate or weak correlations, and results are influenced by population characteristics and methodological approaches [11]. Discrepancies in the reported correlation coefficients between pain intensity and HRQoL measures, such as the SF-36, exist across studies and populations, highlighting the need for context-specific research [11].

In low- and middle-income countries, the burden of musculoskeletal disorders, including neck pain, is increasing [12]. Occupational risk factors such as sedentary work, poor ergonomics, and prolonged use of technology contribute significantly to the prevalence of neck pain in these settings [12]. For instance, medical students often experience neck pain due to rigorous academic and clinical demands [13]. A study in Bangladesh among dental practitioners reported a high prevalence of neck pain, attributing it to inflexible and stressful work postures [14]. Despite this growing burden, robust data on the impact of neck pain on HRQoL, particularly in countries like Bangladesh, remains limited [12].

This lack of localized, comprehensive research indicates a critical gap in the existing literature. There is limited evidence specifically addressing the relationship between pain intensity and multidimensional quality of life among patients with CNP in Bangladesh, utilizing validated instruments such as the visual analog scale (VAS) and SF-36 [12]. Such context-specific data are crucial for understanding the local burden and informing targeted public health interventions. Therefore, this study aimed to assess the relationship between pain intensity and HRQoL in patients with CNP.

## Materials and methods

Study design and setting

A cross-sectional study was conducted in a hospital setting at the Department of Physical Medicine and Rehabilitation of Bangabandhu Sheikh Mujib Medical University in Dhaka, Bangladesh. Data collection spanned 10 months, running from April 2021 through January 2022.

Study population

The study participants were adult patients with a clinical diagnosis of CNP who visited the outpatient department of physical medicine and rehabilitation during the study period. CNP was defined as pain in the cervical region lasting for three months or longer. Adult patients of either sex, aged 20 to 60 years, who were cognitively able to understand and respond to the questionnaire were considered eligible for inclusion. Patients were included irrespective of the presumed clinical cause of CNP, provided they met the eligibility criteria. The study was not designed to identify or confirm a specific anatomical pain generator. Patients were excluded if they had a previous history of cervical spine fracture or cervical spine surgery, shoulder joint pathology, inflammatory or rheumatologic disease, or if they declined to participate or provide informed consent.

Sampling procedure

A non-probability consecutive sampling technique was applied to recruit participants. Throughout the study period, every patient visiting the outpatient department who potentially satisfied the study criteria underwent consecutive assessment. Their eligibility was verified against the pre-established inclusion and exclusion criteria. Individuals who met these requirements and provided written informed consent were enrolled and continued until the target sample size was achieved.

Sample size determination

To calculate the necessary number of participants, the single population proportion formula was applied:

\[n = \frac{Z^2p(1-p)}{d^2}\]

In this equation, Z was set to 1.96, corresponding to a 95% confidence level; p was set to 0.513, based on the previously documented prevalence of neck pain in Bangladesh [15]; and the margin of error (d) was fixed at 0.10. This computation yielded a minimum requirement of 96 participants, and accordingly, 96 individuals were ultimately recruited and analyzed in the study.

Data collection procedures

Data were gathered via face-to-face interviews conducted with a pretested, semi-structured questionnaire. Individuals attending the outpatient department first underwent a clinical evaluation that involved recording their history, conducting a physical examination, and, where clinically warranted, ordering relevant investigations, including blood tests and imaging. Once eligibility was established, each participant received an explanation of the study's aims and procedures, and written informed consent was secured prior to enrollment. Details covering sociodemographic profiles and clinical variables, including how long symptoms had persisted and any coexisting conditions, were captured in a structured format. Pain severity was documented during the interview, and HRQoL was measured with the Bangla version of the SF-36 questionnaire. To ensure consistency and reliable data, all interviews and data collection activities were conducted by the investigators, and completed questionnaires were thoroughly checked for completeness before entering the data.

Sociodemographic and clinical variables

Through interviews, participants provided details on their age, sex, level of education, occupation, monthly household income, and how long they had experienced neck pain. Their height and weight were measured, and these values were used to compute BMI. Participants were subsequently grouped into BMI categories following the criteria established by the World Health Organization [16]. The original study protocol did not include a fibromyalgia-specific tender-point examination or assessment using validated fibromyalgia diagnostic criteria. Concomitant pain at other anatomical sites, including low back pain, was also not systematically recorded.

Pain intensity assessment

The VAS, a widely used tool for gauging how severe a person perceives their pain, was used to assess pain intensity. It took the form of a horizontal 10-cm line on which one end (0) denoted the absence of pain and the other end (10) denoted the most severe pain a person could imagine [17]. Participants were instructed to place a mark at the point along the line corresponding to their pain level at that moment. This marked location was then translated into a numerical value for analysis.

HRQoL

HRQoL was assessed using an interviewer-administered Bangla-translated version of the SF-36 [18]. The standard four-week recall period was applied to items referring to recent health and functioning. The instrument assesses eight domains: physical functioning (PF), role limitation due to physical health (RP), bodily pain (BP), general health (GH), vitality (VT), social functioning (SF), role limitation due to emotional problems (RE), and mental health (MH). Item responses were coded and transformed to a 0-100 scale, and each domain score was calculated by averaging its constituent items. For the present analysis, a study-defined physical health summary (PHS) was calculated as the unweighted arithmetic mean of PF, RP, BP, and GH, and a study-defined mental health summary (MHS) as the unweighted arithmetic mean of VT, SF, RE, and MH. These were descriptive composite measures rather than norm-based SF-36 physical and mental component scores. Higher domain and composite scores indicated better HRQoL.

Statistical analysis

Prior to analysis, the data were verified for completeness and consistency and subsequently entered into SPSS Statistics version 25 (IBM Corp. Released 2017. IBM SPSS Statistics for Windows, Version 25.0. Armonk, NY: IBM Corp.). Continuous variables were presented as means with standard deviations, while categorical variables were reported as frequencies and percentages. To assess the distribution of continuous variables, the Shapiro-Wilk test was applied. Since the variables of interest satisfied the assumptions for parametric testing, Pearson's correlation coefficient was used to examine the relationship between pain intensity, measured via the VAS, and HRQoL, measured using the SF-36 domain and component scores. The strength of each correlation was classified as very weak, weak, moderate, strong, or very strong based on the correlation coefficient. Every test was two-tailed, and results were deemed statistically significant when the p-value fell below 0.05.

Ethical considerations

Before data collection commenced, ethical approval was granted by the Institutional Review Board of Bangabandhu Sheikh Mujib Medical University (approval number: BSMMU/2021/3444). All study procedures adhered to the ethical standards set out in the Declaration of Helsinki. Ahead of enrollment, every eligible participant was fully briefed on the purpose and methods of the study, the voluntary nature of taking part, and their entitlement to withdraw at any stage without facing any adverse consequences. Written informed consent was collected from each participant. Confidentiality of personal data was preserved, and anonymity was upheld across all phases of data collection, analysis, and reporting.

## Results

Table 1 summarizes the baseline sociodemographic characteristics of the participants. The mean age of the participants was 38.37 ± 10.28 years, with the largest proportion belonging to the 31-40 years age group, 42 (43.7%), followed by the 20-30 years age group, 24 (25.0%). A clear male predominance was observed, with 71 male participants (74.0%). Regarding educational status, secondary-level education was the most common, reported by 35 (36.5%) participants, followed by primary education in 26 (27.1%) participants. Service holders constituted the largest occupational group, 27 (28.2%), followed by housewives, 22 (22.9%), and businessmen, 20 (20.8%), indicating a diverse occupational distribution. Monthly income was most commonly 20,001-30,000 Bangladeshi taka, reported by 43 (44.8%) participants, followed by >30,000 Bangladeshi taka, reported by 23 (23.9%) participants. In terms of body mass index, over half of the participants had a normal BMI (50, 52.1%), while 25 (26.0%) were overweight and 21 (21.9%) were obese.

**Table 1:** Baseline sociodemographic characteristics (n = 96) Values are presented as n (%). SD: standard deviation, BDT: Bangladeshi taka, BMI: body mass index

Variable	Category	n (%)
Age (years)	20-30	24 (25.0)
	31-40	42 (43.7)
	41-50	18 (18.8)
	51-60	12 (12.5)
	Mean ± SD	38.37 ± 10.28
Sex	Male	71 (74.0)
	Female	25 (26.0)
Education	No formal education	14 (14.6)
	Primary	26 (27.1)
	Secondary	35 (36.5)
	Higher	21 (21.8)
Occupation	Students	13 (13.5)
	Housewife	22 (22.9)
	Service holder	27 (28.2)
	Businessman	20 (20.8)
	Others	14 (14.6)
Monthly income (BDT)	≤10,000	12 (12.5)
	10,001-20,000	18 (18.8)
	20,001-30,000	43 (44.8)
	>30,000	23 (23.9)
BMI (kg/m²)	Normal	50 (52.1)
	Overweight	25 (26.0)
	Obese	21 (21.9)

The clinical characteristics of the participants are presented in Table 2. The majority of patients (59, 61.5%) reported neck pain lasting less than one year, while 37 (38.5%) had symptoms persisting for one year or more. Regarding comorbid conditions, diabetes mellitus was present in 15 (15.6%) participants, hypertension in 10 (10.4%), and lung disease in five (5.2%). It is important to note that some participants had more than one comorbidity.

**Table 2:** Clinical characteristics of participants (n = 96) * Participants may have more than one comorbidity.

Variable	Category	n (%)
Duration of neck pain	<1 year	59 (61.5)
	≥1 year	37 (38.5)
Comorbidities*	Diabetes mellitus	15 (15.6)
	Hypertension	10 (10.4)
	Lung disease	5 (5.2)

The distribution of HRQoL across different SF-36 domains is shown in Table 3. Among the eight domains, the physical functioning domain demonstrated the highest mean score (58.07 ± 14.18), indicating relatively preserved basic physical abilities. In contrast, role limitation due to physical health had the lowest mean score (36.19 ± 22.02), indicating significant impairment in daily role performance related to physical problems. Other domains, including bodily pain (44.06 ± 11.24), general health (43.02 ± 8.53), vitality (41.46 ± 10.75), social functioning (48.69 ± 10.01), role limitation due to emotional problems (43.40 ± 31.76), and mental health (47.21 ± 9.95), also showed moderate reductions.

**Table 3:** SF-36 domain scores Values are presented as mean ± SD. SD: standard deviation, SF-36: 36-Item Short-Form Health Survey

Domain	Mean ± SD
Physical functioning (PF)	58.07 ± 14.18
Role limitation due to physical health (RP)	36.19 ± 22.02
Bodily pain (BP)	44.06 ± 11.24
General health (GH)	43.02 ± 8.53
Vitality (VT)	41.46 ± 10.75
Social functioning (SF)	48.69 ± 10.01
Role limitation due to emotional problems (RE)	43.40 ± 31.76
Mental health (MH)	47.21 ± 9.95

Table 4 presents the correlation between pain intensity and SF-36 domains. A strong negative correlation was observed between pain intensity and physical functioning (r = −0.713, p < 0.001). Moderate negative correlations were found with role limitation due to physical health (r = −0.447, p < 0.001), role limitation due to emotional problems (r = −0.482, p < 0.001), general health (r = −0.356, p < 0.001), and bodily pain (r = −0.348, p = 0.001). Vitality also showed a weak negative correlation (r = −0.300, p = 0.003). However, no statistically significant correlation was observed with social functioning (r = −0.131, p = 0.205) and mental health (r = −0.165, p = 0.109).

**Table 4:** Correlation between pain intensity and SF-36 domains Pearson’s correlation test was used. A p-value <0.05 was considered statistically significant. SF-36: 36-Item Short-Form Health Survey

Domain	r	p-value
Physical functioning (PF)	−0.713	<0.001
Role limitation due to physical health (RP)	-0.447	<0.001
Bodily pain (BP)	-0.348	0.001
General health (GH)	-0.356	<0.001
Vitality (VT)	-0.300	0.003
Social functioning (SF)	-0.131	0.205
Role limitation due to emotional problems (RE)	-0.482	<0.001
Mental health (MH)	-0.165	0.109

The summary scores of SF-36 components are presented in Table 5. The mean PHS score was 45.33 ± 9.21, while the MHS score was 45.19 ± 10.33.

**Table 5:** Summary of SF-36 component scores SD: standard deviation, SF-36: 36-Item Short-Form Health Survey

Component	Mean ± SD
Physical health summary (PHS)	45.33 ± 9.21
Mental health summary (MHS)	45.19 ± 10.33

Significant moderate negative correlations were observed between pain intensity and both PHS (r = −0.420, p < 0.001) and MHS (r = −0.527, p < 0.001). Furthermore, the total SF-36 score also demonstrated a moderate negative correlation with pain intensity (r = −0.550, p < 0.001) (Table 6).

**Table 6:** Correlation of SF-36 components with pain intensity Pearson’s correlation test was used. A p-value <0.05 was considered statistically significant. SF-36: 36-Item Short-Form Health Survey

Component	r	p-value
Physical health summary (PHS)	-0.420	<0.001
Mental health summary (MHS)	-0.527	<0.001
Total SF-36 score	-0.550	<0.001

## Discussion

In the present study, we evaluated the association between pain intensity and HRQoL among patients with CNP. Our findings demonstrated a consistent reduction in SF-36 scores across multiple domains, indicating a multidimensional impairment in quality of life. Among all SF-36 domains, role limitation due to physical health had the lowest mean score, suggesting greater impairment in daily role and work-related functioning. In contrast, basic physical functioning was relatively better preserved. Importantly, pain intensity showed significant inverse associations with several SF-36 domains and both physical and mental component summary scores, although no significant correlations were observed with social functioning and mental health. The observed male predominance may reflect sex-related differences in referral, healthcare-seeking behavior, and access to tertiary specialist care rather than the population distribution of CNP, which may limit the generalizability of the findings.

One of the key findings of this study was the general decline in SF-36 scores spanning several domains, a pattern that aligns with the wider body of research on CNP. De Vries et al. documented that, among patients with nonspecific CNP receiving rehabilitation, scores across every SF-36 domain fell markedly below the reference values for the general population [19]. In a similar vein, Machino et al. found that individuals suffering from neck and shoulder pain recorded lower Physical Component Summary and Mental Component Summary scores than those free of neck pain, which points to the wide-ranging impact of this condition on perceived health [7]. In the current study, the lowest score was in the role limitation due to physical health domain, indicating that everyday activities and work-related functioning are particularly affected. This finding has important clinical implications, given that the majority of participants were of working age in a setting where functional productivity is closely tied to the well-being of both the individual and their family [20].

Results from our study also showed that the strongest inverse relationship was between pain intensity and physical functioning, with a further moderate negative association between pain intensity and the PHS score. These findings are consistent with previous evidence that greater pain severity is linked to poorer functional capacity, movement restriction, and disability in CNP. Asiri et al. found that in patients with CNP, kinesiophobia was strongly correlated with pain intensity and significantly associated with functional performance [21]. Similarly, Saavedra-Hernández et al. demonstrated that pain intensity and pain-related fear were independently correlated with self-reported disability in chronic mechanical neck pain [22]. These findings are biologically and behaviorally plausible. Greater pain intensity may promote fear-avoidance behaviors, leading to reduced cervical mobility, impaired muscle endurance, physical deconditioning, and lower ability to sustain work or routine activities. Rasmussen-Barr et al., in a systematic review, further showed that exercise interventions can improve both pain and disability in CNP, indirectly reinforcing the close relationship between unresolved pain and declining physical function [23].

Another notable finding was the inverse association of pain intensity with role limitation due to emotional problems and the study-defined MHS, despite the absence of a significant correlation with the mental health domain. In this study, MHS was calculated as the unweighted arithmetic mean of vitality, social functioning, role limitation due to emotional problems, and mental health. Among these constituent domains, role limitation due to emotional problems showed the strongest correlation with pain intensity (r = −0.482, p < 0.001). In contrast, vitality showed a weaker correlation, and social functioning and mental health were not significantly correlated with pain intensity. Thus, the association between pain intensity and MHS appears to have been largely carried by the emotional-role domain. Averaging the four constituent domains may also have reduced domain-specific measurement variability and produced a more stable composite estimate. Therefore, the MHS finding should be interpreted as an association with the composite score rather than with the mental health domain alone. Psychological factors, including anxiety, depression, distress, and emotional problems, have been identified as important contributors to the development and persistence of neck pain [24]. Depressive symptoms, distress, and anxiety have also been associated with poorer outcomes among adults with cervical spine pain [25]. Furthermore, a longitudinal Korean cohort study found that CNP was associated with worsening depressive symptoms over time, particularly among individuals with pre-existing depression [26].

The current findings further suggest that pain intensity was moderately and inversely associated with the general health, vitality, and bodily pain domains of the SF-36. This indicates that as pain becomes more severe, patients tend to perceive their health more negatively and experience reduced energy and greater bodily burden. A study of patients with nonspecific CNP similarly reported negative correlations between pain intensity and SF-36 physical summary scores, supporting the view that persistent pain progressively undermines perceived health status [27]. The absence of statistically significant correlations with social functioning and the mental health domain, however, should be interpreted cautiously. These findings do not imply that such dimensions are unaffected; rather, they suggest that they may be shaped by factors beyond pain intensity alone. In this regard, the Saskatchewan Health and Back Pain Survey found that prevalent neck pain was not independently associated with mental HRQoL after adjustment for relevant covariates [28]. It is also possible that social support, cultural coping patterns, or gradual adaptation to chronic symptoms may partially buffer the effects of pain on social participation and emotional well-being. Overall, the present findings add to the growing body of evidence that CNP is a multidimensional condition in which greater pain intensity is associated with poorer HRQoL, particularly in physical and emotionally mediated functional domains.

Several limitations should be acknowledged. The cross-sectional design precluded causal inference, while single-center consecutive sampling may have introduced selection bias and limited generalizability. The male-predominant sample may reflect sex-related differences in referral and healthcare access. Sex-stratified analyses were not performed, and the small female subgroup would have limited the precision of such analyses. Adults older than 60 years were excluded, limiting applicability to older populations. Pain intensity and HRQoL were self-reported, and relevant biopsychosocial factors, including pain-related disability, pain catastrophizing, and fear-avoidance, were not assessed. Fibromyalgia-specific evaluation and documentation of low back pain or other pain sites were also not performed. Finally, only unadjusted correlations were available; therefore, residual confounding cannot be excluded, and the findings should not be interpreted as independent effects of pain intensity on HRQoL. Future studies should include older adults, standardized assessment of fibromyalgia and multisite pain, detailed characterization of potential cervical pain generators, relevant biopsychosocial factors, and prospective evaluation before and after appropriate treatment, including surgery when clinically indicated. The sample size was determined using a prevalence-based formula rather than an effect-size-based calculation for correlation analysis, which may have limited the precision for detecting weaker associations.

## Conclusions

CNP was associated with a substantial reduction in HRQoL across multiple domains. The most pronounced impairment was observed in role limitation due to physical health, indicating that pain predominantly affects patients’ ability to perform daily roles and functional activities. In contrast, basic physical functioning remained relatively less affected. Pain intensity showed significant inverse relationships with several physical and emotion-related domains, as well as overall summary scores. Although social functioning and mental health domains did not show significant correlations, the overall HRQoL declined with increasing pain severity. These findings underscore the importance of comprehensive management strategies that emphasize pain control, restoration of functional capacity, and multidimensional rehabilitation in patients with CNP.
